# Functional characterization of *NBS-LRR* genes reveals an *NBS-LRR* gene that mediates resistance against *Fusarium* wilt

**DOI:** 10.1186/s12915-024-01836-x

**Published:** 2024-02-27

**Authors:** Yunpeng Cao, Wanzhen Mo, Yanli Li, Yao Xiong, Han Wang, Yingjie Zhang, Mengfei Lin, Lin Zhang, Xiaoxu Li

**Affiliations:** 1 CAS Key Laboratory of Plant Germplasm Enhancement and Specialty Agriculture, Wuhan Botanical Garden, Chinese Academy of Sciences, Wuhan, 430074 China; 2 School of Health and Nursing, Wuchang University of Technology, Wuhan, China; 3 Beijing Life Science Academy, Beijing, 102209 China; 4 Hubei Shizhen Laboratory, School of Basic Medical Sciences, Hubei University of Chinese Medicine, Wuhan, 430065 China; 5 Institute of Biological Resources, Jiangxi Academy of Sciences, Nanchang, Jiangxi 330224 China; 6 School of Life Sciences, Anhui Agricultural University, Hefei, China; 7 Forestry College, Central South University of Forestry and Technology, Changsha, 410004 China

**Keywords:** *NBS-LRR*, Tung tree, Functional, VIGS, *Fusarium*

## Abstract

**Background:**

Most disease resistance (R) genes in plants encode proteins that contain leucine-rich-repeat (LRR) and nucleotide-binding site (NBS) domains, which belong to the *NBS-LRR* family. The sequenced genomes of *Fusarium* wilt-susceptible *Vernicia fordii* and its resistant counterpart, *Vernicia montana*, offer significant resources for the functional characterization and discovery of novel *NBS-LRR* genes in tung tree.

**Results:**

Here, we identified 239 *NBS-LRR* genes across two tung tree genomes: 90 in *V. fordii* and 149 in *V. montana*. Five *VmNBS-LRR* paralogous were predicted in *V. montana*, and 43 orthologous were detected between *V. fordii* and *V. montana*. The orthologous gene pair *Vf11G0978-Vm019719* exhibited distinct expression patterns in *V. fordii* and *V. montana*: *Vf11G0978* showed downregulated expression in *V. fordii*, while its orthologous gene *Vm019719* demonstrated upregulated expression in *V. montana*, indicating that this pair may be responsible for the resistance to *Fusarium* wilt in *V. montana*. *Vm019719* from *V. montana*, activated by *VmWRKY64*, was shown to confer resistance to *Fusarium* wilt in *V. montana* by a virus-induced gene silencing (VIGS) experiment. However, in the susceptible *V. fordii*, its allelic counterpart, *Vf11G0978*, exhibited an ineffective defense response, attributed to a deletion in the promoter’s W-box element.

**Conclusions:**

This study provides the first systematic analysis of *NBS-LRR* genes in the tung tree and identifies a candidate gene that can be utilized for marker-assisted breeding to control *Fusarium* wilt in *V. fordii*.

**Supplementary Information:**

The online version contains supplementary material available at 10.1186/s12915-024-01836-x.

## Background

Tung tree, belonging to the Euphorbiaceae family, are important woody oil-producing trees in China. Tung oil produced from tung tree seeds is rich in the trivalent unsaturated fatty acid eleostearic acid [1, 2], which has excellent properties such as corrosion resistance and acid and alkali resistance. *Vernicia fordii* and *Vernicia montana* represent the two principal cultivars in China. Compared to *V. montana*, the seeds of *V. fordii* exhibit a more rapid maturation process and yield superior oil quality. However, the recent surge in *Fusarium* wilt occurrences has adversely affected the cultivation and industrial development of tung tree. Previous studies have revealed that *V. fordii* is susceptible to *Fusarium* wilt, while *V. montana* exhibits effective resistance to this disease [3, 4]. At present, there is no established cure for *Fusarium* wilt in *V. fordii*. The most effective strategy for managing this disease involves using disease-resistant *V. montana* as the rootstock and grafting it with *V. fordii* as the scion. Consequently, it is imperative to explore the disease resistance mechanisms in *V. montana* and understand the factors that contribute to the susceptibility of *V. fordii* to *Fusarium* wilt.

Plants encode multiple disease-resistance (*R*) genes that confer resistance to insects and pathogens [3–5]. In the past few decades, researchers have cloned over 300 *R* genes from plants [6]. The proteins encoded by these *R* genes exhibit diverse domain combinations [6, 7]. Among these domains, the nucleotide-binding site (NBS) and leucine-rich repeat (LRR) domains are the most widespread in known *R* genes, collectively referred to as *NBS-LRR* genes [8]. The *NBS-LRRs* originate from green plants [9], and their encoded proteins facilitate plant resistance primarily by recognizing receptors within pathogens themselves [10]. Considering the variations in their N-termini structures, *NBS-LRRs* can be further categorized into two types: Toll/interleukin-1 receptor (TIR)-NBS–LRR (TNL) types, characterized by a TIR domain, and non-TIR-NBS-LRR (non-TNL) types, which feature either leucine zipper (LZ) or coiled-coil (CC) domains in place of the TIR domain [8, 11, 12]. The C-terminal LRR domains of NBS-LRR proteins are crucial for protein-protein interactions and pathogens recognition specificity [13, 14]. Conversely, the N-terminal NBS domains bind GTP and ATP facilitating hydrolytic reactions that provide energy for downstream signaling processes [13, 14]. *NBS-LRRs* primarily confer disease resistance in plants by recognizing pathogens. For example, the NBS-LRR protein RPS5 is capable of identifying bacteria expressing the type III effector AvrPphB, thereby conferring resistance to downy mildew [15]. An NBS-LRR protein located at the *Rpp1* locus counteracts the effectiveness of *Rpp1*-mediated resistance to *Phakopsora pachyrhizi* in soybean [16]. Through rapid evolution, *R* genes enable plants to detect avirulence genes in various pathogens, triggering downstream signaling cascades that culminate in programmed cell death, hypersensitive reactions, and defense responses [17–20].

Recent advancements in sequencing technologies have facilitated the identification of *NBS-LRR* genes across a broad spectrum of plant species, such as Arabidopsis, rice, cabbage, grape, and sunflower [12, 21–24]. These investigations have revealed variations in the size of the N*BS-LRR* family across different plant genomes. Here, we determined members of the *NBS-LRR* family and conducted a comparative analysis across the genomes of *V. fordii* and *V. montana*. Furthermore, we carried out functional analyses to elucidate the roles of these genes in *Fusarium* wilt resistance between *V. montana* and *V. fordii*. As a result, we established a potential contribution of *VmNBS-LRR* to the resistance of *V. montana*’s resistance to *Fusarium* wilt. These findings provide crucial insights into *Fusarium* wilt-responsive *NBS-LRRs*, which can serve as pivotal targets for molecular breeding aimed at enhancing disease resistance in tung tree.

## Results and discussion

### *NBS–LRRs* in *V. montana* and *V. fordii*

Utilizing HMMER software for analysis, we identified a combined total of 239 NBS-containing sequences in the two *Vernicia* species: 90 in *V. fordii* and 149 in *V. montana*. From the *V. fordii NBS-LRRs*, *90 VfNBS-LRRs* were categorized into four subgroups: CC-NBS-LRR (12), NBS-LRR (12), CC-NBS (37), and NBS (29) (Table 1 and Additional file 1). Notably, 49 VfNBS-LRRs from *V. fordii* contained the CC domain, accounting for 54.4% of the VfNBS-LRRs. It is important to highlight that no TIR domains were found in VfNBS-LRRs, indicating that none of the resistance genes in *V. fordii* belonged to the TIR class. This is consistent with previous studies that have shown the absence of TIR domain-containing NBS-LRRs in monocots, whereas they are more commonly observed in eudicots [10]. In fact, the loss of NBS-LRRs containing TIR domains in eudicots has only been reported in *Sesamum indicum* [25] and is now also observed in *V. fordii* as reported in this study. However, among the 149 *VmNBS-LRRs* identified in *V. montana*, they were divided into seven subgroups: CC-NBS-LRR (9), TIR-NBS-LRR (3), CC-TIR-NBS (2), TIR-NBS (7), NBS-LRR (12), CC-NBS (87), and NBS (29) (Table 1 and Additional file 2). Among them, 98 *VmNBS-LRRs* contained the CC domains, accounting for 65.8% of the *VmNBS-LRRs*, 12 *VmNBS-LRRs* possessed the TIR domains (8.1%), and 2 *VmNBS-LRRs* contained both the CC and TIR domains. These findings provide valuable insights into the evolutionary aspects of plant disease-resistance genes.

**Table 1:** Classification of *NBS-LRR* genes in *V. fordii* and *V. montana*

**Types**	**Number**	
	***V. fordii***	***V. montana***
CC-NBS-LRR	12	9
TIR-NBS-LRR	0	3
**NBS-LRR truncated NBS-LRR**		
CC-TIR-NBS	0	2
TIR-NBS	0	7
NBS-LRR	12	12
CC-NBS	37	87
NBS	29	29
**Total NBS**	90	149
Total NBS with LRR	24	24
Total NBS without LRR	66	125

The LRR domain is vital for plant immune responses, facilitating both protein-ligand and protein-protein interactions [26–28]. In this study, we identified two distinct LRR domains, LRR3 and LRR8, among the 90 VfNBS-LRRs of *V. fordii*, with 4 and 23 numbers, respectively (Additional file 3). Conversely, *V. montana* displayed four types of LRR domains: LRR1 (2), LRR3 (4), LRR4 (2), and LRR8 (20) (Additional file 4). The LRR1 domain was exclusive to the NL and CNL proteins of *V. montana*, while the LRR4 domain was only present in the NL proteins of *V. montana*. Interestingly, these two LRR domains were not found in *V. fordii*, indicating the occurrence of LRR domain loss events in *V. fordii* during evolution.

### Chromosomal distributions and evolutionary analysis of *NBS-LRRs*

**Fig. 1:**
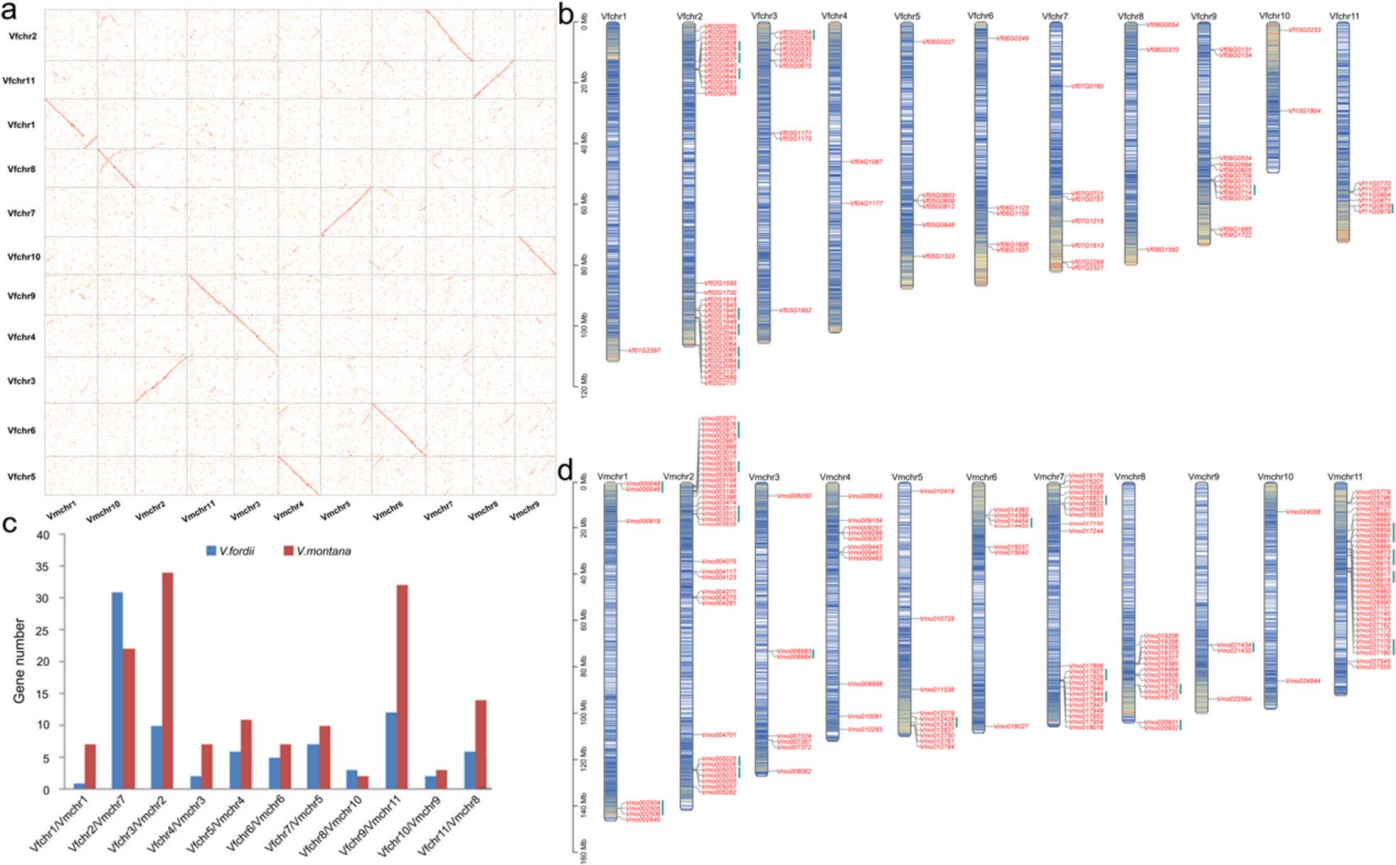
Chromosomal distribution analysis of *NBS-LRR* genes from *Vernicia*. **a** Analysis of homologous chromosomes between *V. fordii* and *V. montana*. **b ***VfNBS-LRR* genes were mapped on the chromosomes of *V. fordii*. **c** Comparison of the location of *NBS–LRRs* on each chromosome between *V. fordii* and *V. montana*. **d ***VmNBS-LRR* genes were mapped on the chromosomes of *V. montana*

The genomes of *Vernicia* species, comprising 11 chromosomes [2, 29], are designated as Vfchr.1-Vfchr.11 in *V. fordii* and Vmchr.1-Vmchr.11 in *V. montana*. To investigate the chromosomal distribution of *NBS-LRR* genes in these species, we first analyzed the homologous chromosome relationships between their genomes (Fig. 1a). One-to-one syntenic relationships were observed, such as Vfchr1 in *V. fordii* corresponded to Vmchr1 in *V. montana*, and Vfchr2 and Vfchr3 respectively aligned with Vmchr7 and Vmchr2 (Fig. 1a). Subsequently, we found significant differences in the distributions of *NBS-LRR* genes across these chromosomes between the two species (Fig. 1b–d). In *V. fordii*, a higher number of *VfNBS-LRRs* were located on Vfchr2, Vfchr3, and Vfchr9, while a lower number was found on Vfchr1, Vfchr4, and Vfchr10 (Fig. 1b, c). Similarly, in *V. montana*, a higher number of *VmNBS-LRRs* were present on Vmchr2, Vmchr7, and Vmchr11, while a lower number was observed on Vmchr9 and Vmchr10 (Fig. 1c, d). These results suggest that *NBS-LRR* genes are distributed non-randomly across all chromosomes, showing a clustered distribution. The enrichment of *NBS-LRRs* in corresponding genomic regions suggested that the evolution of resistance genes may involve tandem duplications of linked gene families, as reported by previous studies [8, 30].

**Fig. 2:**
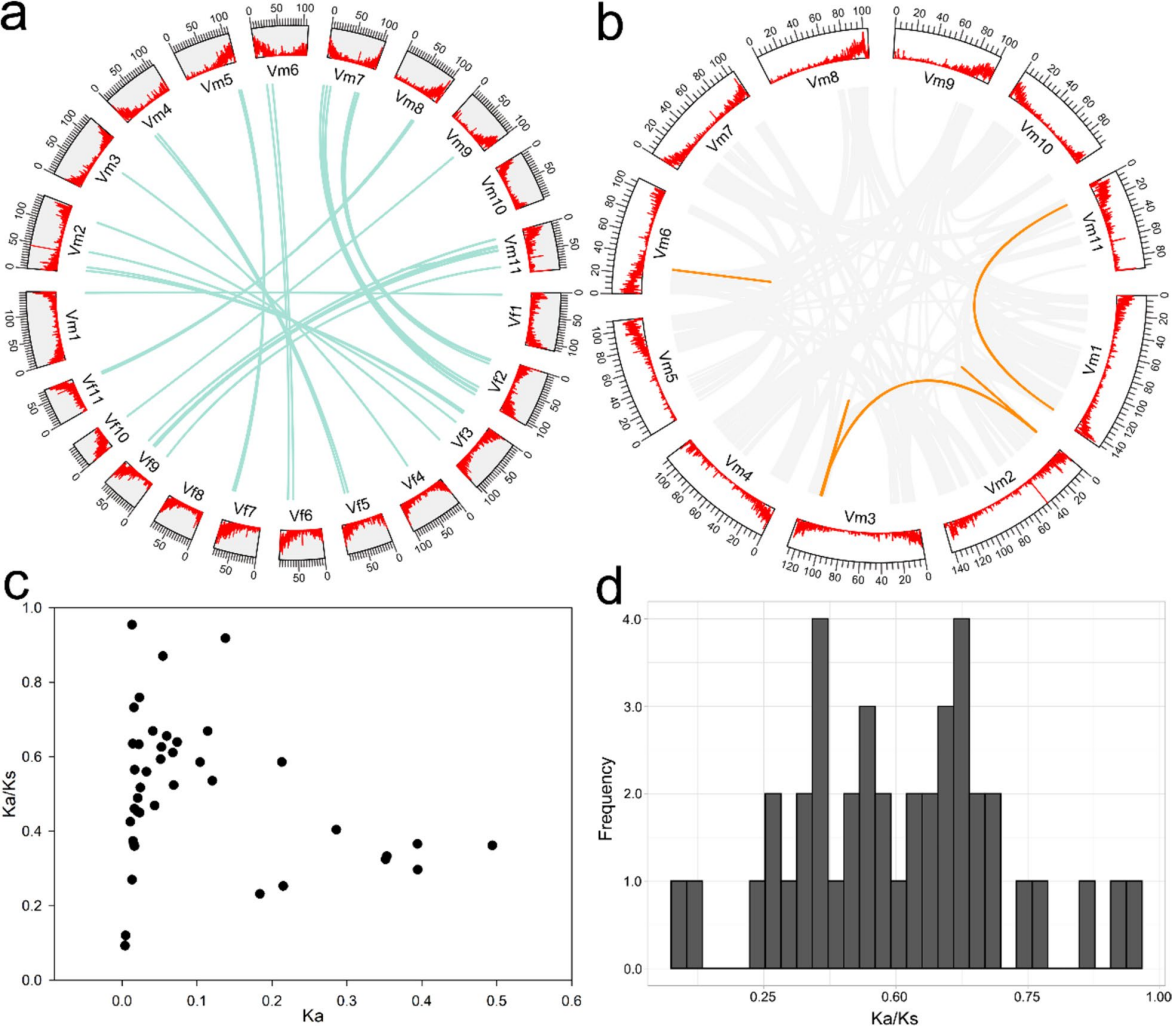
Gene duplication and environmental selection pressure analysis. **a** One-to-one orthologous *NBS-LRR* genes between *V. fordii* and *V. montana*. The green lines indicate orthologous *NBS-LRR* genes in a similar homologous chromosomal location between *V. fordii* and *V. montana*. **b** Gene duplication analysis of *V. montana*. The orange lines indicate *NBS-LRR* duplication genes in *V. montana*. **c** Scatter plots of the Ka/Ks ratios of orthologous *NBS-LR*R gene pairs between *V. fordii* and *V. montana*. The X-axes and Y-axes indicate the Ka and Ka/Ks ratio for each pair, respectively. **d** Frequency distribution histogram of Ka/Ks of orthologous *NBS-LRR* gene pairs between *V. fordii* and *V. montana*

In general, *NBS-LRRs* can be divided into various evolutionary groups based on the differences in their structural domains [12, 31]. In this study, we observed that *V. fordii* exhibited a smaller number of LRR domains compared to *V. montana*. Additionally, *V. montana* displayed a wider variety of LRR domains within its VmNBS-LRRs relative to VfNBS-LRRs. To investigate the relationship between *NBS-LRRs* in *V. montana* and *V. fordii*, we detected one-to-one orthologous gene pairs between these two sister species of *Vernicia* (Fig. 2a and Additional file 5). Totally, 43 one-to-one orthologous gene pairs were identified, and most of these pairs were located on corresponding sister chromosomes in the *Vernicia* genomes. Furthermore, we compared the domains and groups of the orthologous *NBS-LRRs* genes and found that some *CNL* sequences were orthologous genes with *TNL* genes. In *Arachis*, three *TNL* genes were identified within the CNL group, while one *TNL* gene was found clustering within the *CNL* gene [31]. Similar observations have been reported in other plant species such as *Eucalyptus grandis*, *Vitis vinifera*, and *Medicago truncatula*, where *TNL* genes are integrated within the CNL group [32–34]. These findings suggest that the recombination events have occurred within the NBS domain. For example, Innes et al. [35] confirmed that recombination occurred between some NBS domains from TNL and CNL sequences.

### Gene duplication and environmental selection pressure analysis

Plants have undergone one or multiple polyploidization events during their long evolutionary history [36–38]. Tung tree has also experienced at least one polyploidization event(s) [2, 29]. To delve deeper into the impact of polyploidization on *NBS-LRRs* in *V. montana* and *V. fordii*, we assessed the intraspecific collinearity of their genomes using the MCScanX software [39]. This approach facilitated the identification of paralogous genes within the *NBS-LRRs* of *V. montana* and *V. fordii*, as these genes have the potential to generate novel resistant functions compared to ancestral genes [40]. Similar to *NBS-LRRs* found in other plants [21, 23, 24], the *NBS-LRRs* in the *Vernicia* genomes have undergone many tandem duplication events. Specifically, we identified a total of 10 tandemly duplicated *NBS-LRR* sequences in the *V. fordii* genome (Fig. 1b), and 20 tandemly duplicated *NBS-LRR* sequences in the *V. montana* genome (Fig. 1d). Chromosomes 2 in *V. fordii* and chromosomes 2 and 11 in *V. montana* exhibited the highest number of tandemly duplicated genes.

In domesticated plant species, duplication events have been observed to occur more frequently [41]. Segmental duplication events have significantly contributed to neo-functionalization or sub-functionalization processes, resulting in the acquisition of novel and distinct functions in comparison to the ancestral genes [42]. The expansion of *NBS-LRR* genes may be regarded as a plant-specific adaptation to extracellular signal perception [42, 43], such as the ability to recognize various PAMPs in Arabidopsis [44], given the abundance of pathogens in *Vernicia* [3, 45]. Further research revealed that five *NBS-LRR* gene pairs in *V. montana* (Fig. 2b and Additional file 6) likely originated from a duplication event, whereas no duplication events were detected in the *VfNBS-LRR* genes. These results indicate that species differentiation occurred after the divergence of *V. montana* and *V. fordii* from a common ancestor, and then the gene loss events occurred in *VfNBS-LRRs* of *V. fordii* during the long-term evolution process, which finally led to the *V. fordii* containing fewer members of the *NBS-LRR* family than the *V. montana*. These findings may offer an explanation for the resistance of *V. montana* to *Fusarium* wilt, while *V. fordii* remains susceptible to the disease.

In this study, we calculated the Ka/Ks values of all *NBS-LRR* gene pairs to investigate the evolutionary constraints acting on these genes. The results, as presented in Additional file 7, suggest that the Ka/Ks values of *NBS-LRR* gene pairs in *V. montana* were less than one, indicating a purifying selection on these gene pairs in *V. montana*. The number of duplicated pairs varied between *V. montana* and *V. fordii*, with the former exhibiting strong purifying selection and showing slow protein-level evolution. Subsequently, we compared orthologous gene pairs between *V. montana* (resistant to *Fusarium* wilt) and *V. fordii* (susceptible to *Fusarium* wilt) to identify disease resistance-related loci. The ratio of Ka to Ks was calculated for each *NBS-LRR* gene pair to determine the selective pressure on them (Additional file 8 and Fig. 2c). The peaks at Ka/Ks ratio of 0.3–0.5 and 0.6–0.7 (Fig. 2d) were observed indicating purifying selection between *V. montana* and *V. fordii* (Ka/Ks < 1). Previous studies on cultivated and wild *Vigna angularis* have also shown similar patterns, with peaks at Ka/Ks ratios of 0.4–0.6 and 0.6–0.9, suggesting the presence of novel disease resistance alleles in wild *V. angularis* that differ from those in the cultivated *V. angularis*, such as *Vang02g14420*, *Vang0229s00140*, *Vang0291s00070*, and *Vang03g15160* [46].

### Expression patterns of differentially expressed *NBS-LRRs* after infection of *Fusarium* wilt

The *NBS-LRR* family represents a widespread and ancient group of disease-resistance genes that play key roles in safeguarding plants against various pathogens [47]. The number of *NBS-LRRs* differs significantly between *V. montana* and *V. fordii*, with 149 and 90, respectively. Furthermore, only 33.8% of the *NBS-LRRs* were identified as orthologs between *V. montana* and *V. fordii*. Despite the similarity in genomic features between the two species [2, 29], this discrepancy in *NBS-LRR* counts suggests that they might have undergone gene gains or losses to adapt to distinct environments [30, 48]. This divergence might also explain the contrasting susceptibility of *V. fordii* to *Fusarium* wilt and the resistance of *V. montana* to this disease [3, 4, 49].

**Fig. 3:**
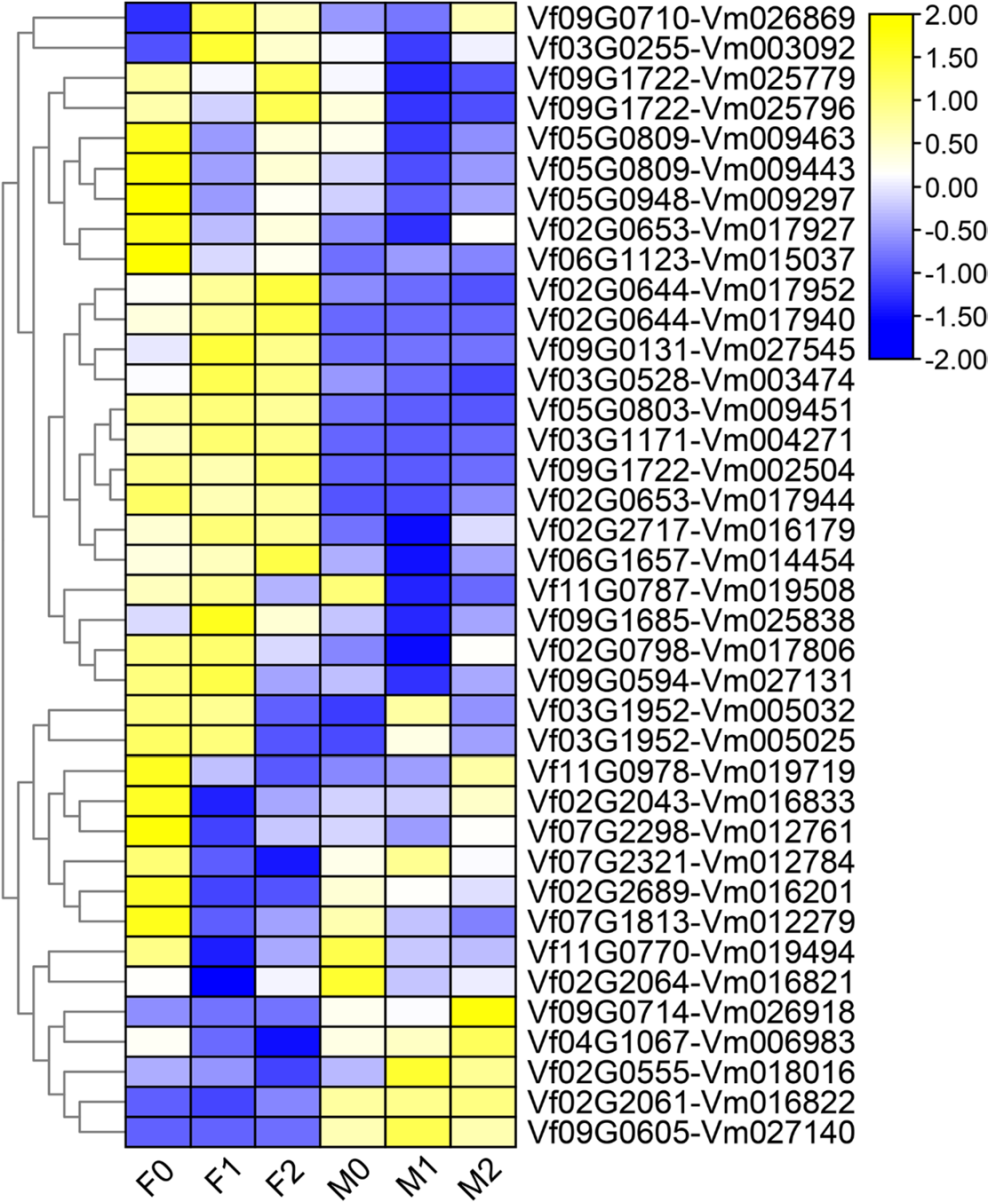
Expression of one-to-one orthologous *NBS-LRR* genes in root tissue of *V. fordii* and *V. montana* under *Fusarium* wilt infection. The RNA-seq data obtained from Chen et al. [45]. The heat map depicts expression profiles of one-to-one orthologous *NBS-LRR* genes in *V. fordii* (left) and *V. montana* (right) in response to *Fusarium* wilt during various infection stages: 0, uninfected stage; 1, 2 days after *Fusarium* wilt infection (dpi); 2, 8 dpi. F0–F2 indicated the expression of *VfNBS-LRRs* in *V. fordii* during various infection stages (0, 1, 2) by the pathogen *Fusarium* wilt; M0-M2 indicated the expression of *VmNBS-LRRs* in *V. montana* during the infection stage (0, 1, 2) by the pathogen *Fusarium* wilt

To determine the mechanism of *NBS-LRRs* in tung tree infected with *Fusarium* wilt, we further detected the expression levels of these genes during various infection stages in *V. fordii* and *V. montana* [45]. Among 43 orthologous *NBS-LRR* gene pairs in these two *Vernicia* species, five gene pairs were not detectable at each stage response to wilt disease. Specifically, we further focused on the expression patterns of the remaining 38 orthologous *NBS-LRR* gene pairs in response to wilt disease. Figure 3 displays the results, revealing divergent expression patterns among most *NBS-LRR* genes. For example, genes such as *Vf09G1722*, *Vf02G0644*, and *Vf02G0653* were highly expressed in the roots of *V. fordii* uninfected and infected with *Fusarium* wilt, while their corresponding orthologous genes exhibited either low expression or were not expressed at all in *V. montana*. Notably, we found that one orthologous gene pair *Vf11G0978-Vm019719* exhibited consistent upregulated expression in *V. montana*, in contrast to persistent downregulation in *V. fordii*. These results suggest that this gene pair may be responsible for the resistance to *Fusariu*m wilt in *V. montana* and the susceptibility to *Fusarium* wilt in *V. fordii*.

### *Vm019719* positively regulates the resistance to *Fusarium* wilt in *V. montana*

**Fig. 4:**
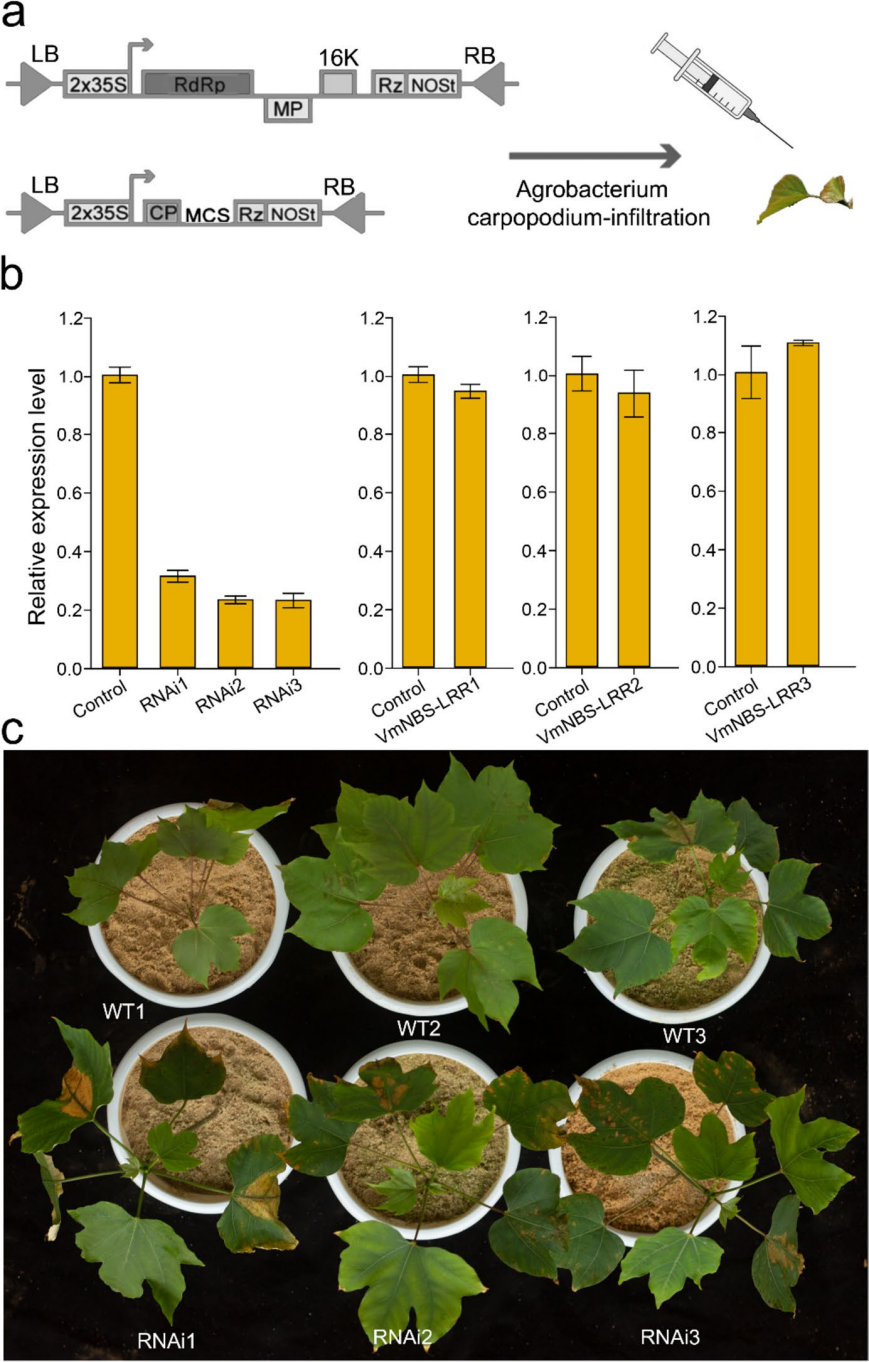
Silencing of *Vm019719* reduces *Fusarium* wilt resistance in *V. montana*. **a** Diagram of the VIGS technique used for infecting the *V. montana* leaves. **b** The silencing efficiency of the *Vm019719* using qRT-PCR. Standard deviations (SDs) were calculated from three biological replicates. **c** Phenotypic identification of *Fusarium* wilt resistance in *V. montana* after silencing of *Vm019719*

Pathogen attack by *Fusarium* wilt induced the expression of *Vm019719* in *V. montana* but cannot induce the expression of its allele *Vf11G0978* in *V. fordii* (Fig. 3). Amino acid sequence alignment revealed that the sequences were basically identical to the allele *Vm019719*, except for a non-functional region (i.e., excluding NBS, TIR, CC and LRR domains) at the N-terminal insertion end of *Vf11G0978*. The primers used in the experiment are listed in Additional file 9. To further investigate the role of *Vm019719* in disease resistance, we employed the VIGS method [50] to downregulate its expression in *V. montana*. The qRT-PCR analysis confirmed the successful downregulation of *Vm019719* expression in the VIGS plants, while the expression remained unchanged in the control plants (Fig. 4a, b). To verify whether the paralogs of *Vm019719* were cross-silenced, we selected three genes (*VmNBS-LRR1: Vm016833*, *VmNBS-LRR2: Vm012761*, and *VmNBS-LRR3: Vm026869*) that had the closest expression pattern to *Vm019719* for evaluation (Fig. 4b). The expression of these selected paralogs was unaffected in *Vm019719* VIGS plants, as revealed by qRT-PCR, indicating the specific downregulation of *Vm019719* by VIGS. To assess the potential attenuation of disease resistance *V. montana* RNAi *Vm019719*, three transgenic lines, RNAi1, RNAi2, and RNAi3, and control plants were selected and placed under *Fusarium* wilt challenge (Fig. 4c). The RNAi plants displayed typical symptoms of *Fusarium* wilt, including necrosis, chlorosis, and wilting, whereas the control *V. montana* plants exhibited significant resistance to the disease with only minor symptoms.

### *WRKY* conferred disease resistance to *Fusarium* wilt in tung tree

**Fig. 5:**
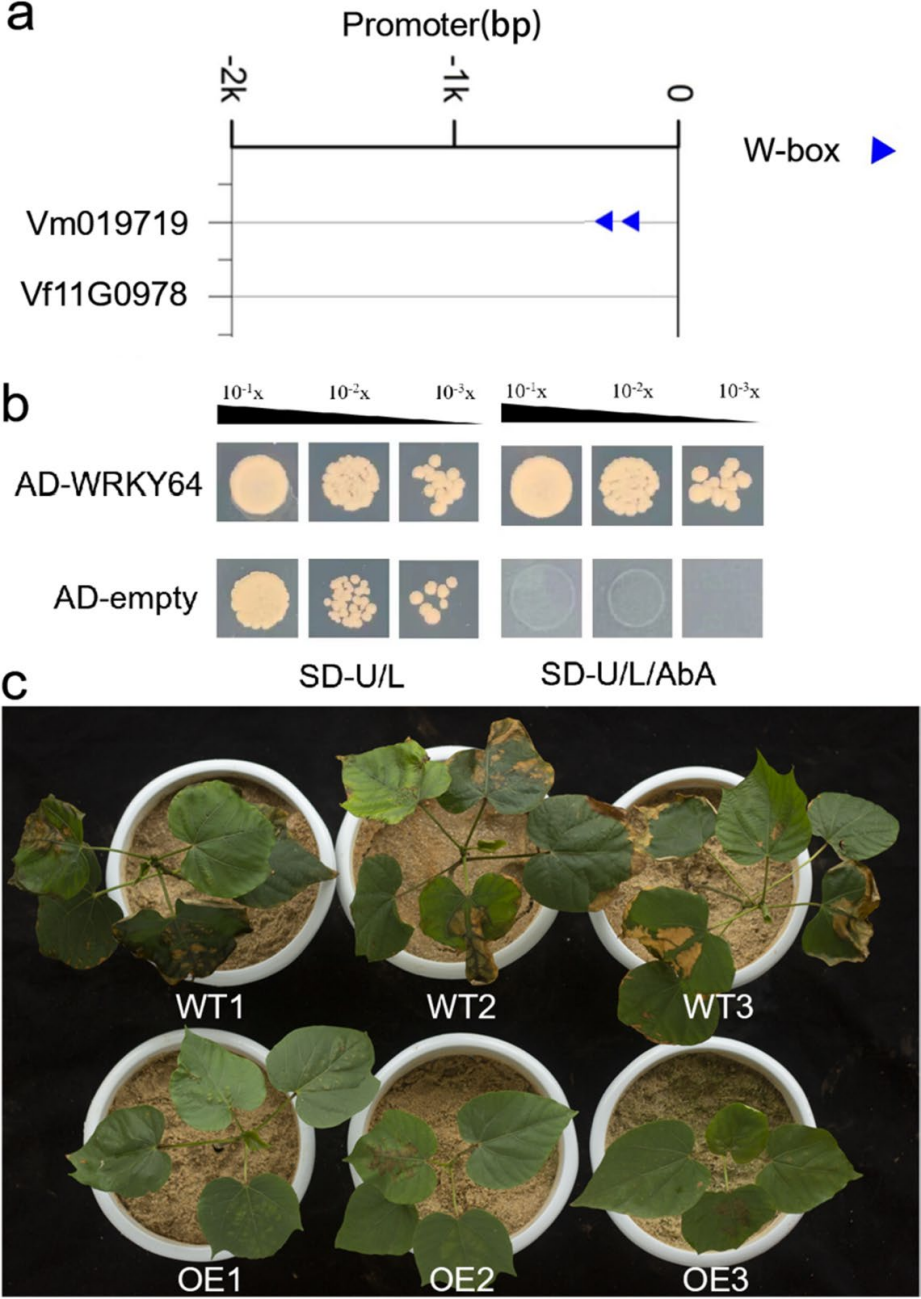
Overexpression of *Vm019719* driven by *Vm019719* promoter enhances *Fusarium* wilt resistance in *V. fordii*. **a** Promoter *cis*-acting element analysis of *Vf11G0978* and *Vm019719*. **b** WRKY64 directly bound to the *Vm019719* promoter. **c** Overexpression of *Vm019719* driven by *Vm019719* promoter enhances *Fusarium* wilt resistance in *V. fordii*

Since the CDSs of *Vm019719* and *Vf11G0978* were basically identical, we cloned their promoter sequences from the genomes of *V. montana* and *V. fordii*, respectively. Subsequently, we constructed AD bait vectors respectively and identified their upstream regulatory transcription factors from *V. fordii* and *V. montana* yeast libraries. Interestingly, we identified a potential regulator, WRKY64 (Vm005641), from the *V. montana* library, but no corresponding transcription factor was screened in another yeast library. Promoter sequence analysis showed that the promoter of *Vm019719* (ProVm019719) had two conserved WRKY binding W-box elements (C/T)TGAC(T/C) [51–53], while ProVf11G0978 had no corresponding elements (Fig. 5a). WRKY64, an ortholog of the Arabidopsis transcription factor WRKY70 [54], has been reported to positively regulate defense against *Fusarium* infection [53, 54]. To confirm the direct binding of WRKY64 to the *Vm019719* promoter and its impact on the transactivation of *Vm019719*, we performed a yeast single-hybrid experiment. The results confirmed that WRKY64 can indeed bind to the promoter of *Vm019719* to activate its expression (Fig. 5b). Furthermore, we overexpressed the *Vm019719* using its own promoter, which led to enhanced resistance against *Fusarium* wilt in *V. fordii*. As depicted in Fig. 5c, the control plants exhibited typical symptoms of *Fusarium* wilt infection, including necrosis, chlorosis, and wilting, while the overexpression *V. fordii* plants were significantly resistant to this *Fusarium* wilt disease by showing only minor symptoms. Taken together, these results demonstrate that WRKY64 can indeed combine with the promoter of *Vm019719* to regulate its expression, thereby providing tung trees with the capacity to resist *Fusarium* wilt.

### A proposed mechanism for *V. montana*’s resistance to *Fusarium* wilt

**Fig. 6:**
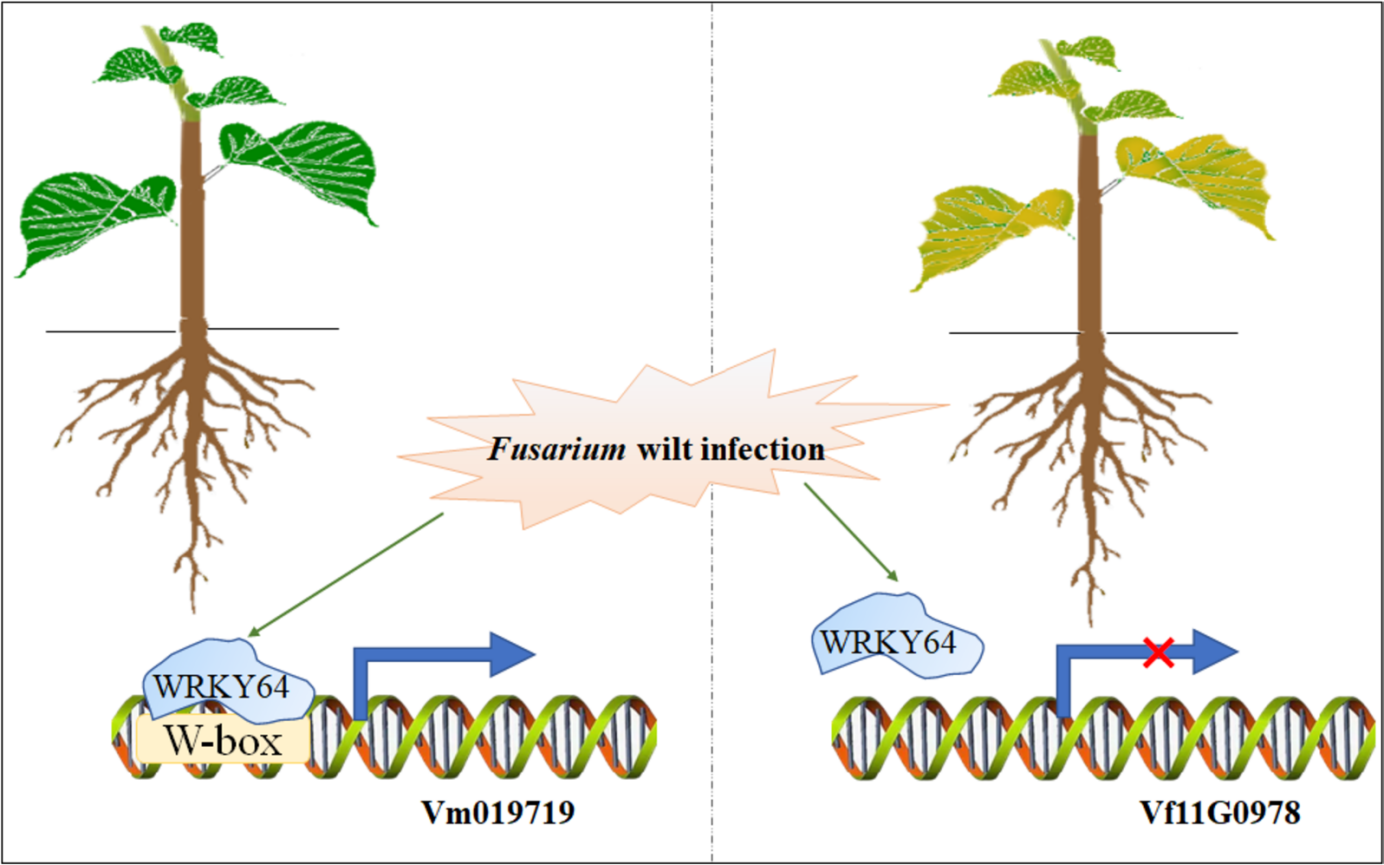
The mechanism of *V. montana* significantly resistant to *Fusarium* wilt. Upon *Fusarium* infection, the expression level of *Vm019719* rapidly increases in *V. montana*, which is driven by WRKY64

Through our examination of the genomic characteristics of the *Fusarium* wilt-susceptible *V. fordii* and *Fusarium* wilt-resistant *V. montana*, we successfully identified the presence of *NBS-LRR* genes. Among these genes, the allele pair *Vm019719-Vf11G0978* stood out as potential key players in conferring resistance to *Fusarium* wilt in tung tree. Notably, *Vm019719-Vf11G0978* displayed downregulated expression in *V. fordii* but showed upregulated expression in *V. montana*. Extending our analysis, we conducted various experiments including *cis*-acting element analysis, VIGS, overexpression studies, and yeast one-hybrid experiments. This comprehensive investigation led us to unveil a novel disease defense mechanism, wherein the transcription factor WRKY64 binds to the W-box elements to effectively defend against *Fusarium* wilt infection (as depicted in Fig. 6).

## Conclusions

In this study, we identified *NBS-LRRs* in two tung tree genomes: *Fusarium wilt*-susceptible *V. fordii* and *Fusarium* wilt-resistant *V. montana*. Interestingly, we observed a preferential loss of LRR domains in *V. fordii* compared to *V. montana*. The expression patterns of *NBS-LRRs* indicated their potential contributions to disease resistance in tung tree. Notably, the orthologous gene pair *Vf11G0978-Vm019719* displayed consistent downregulated expression in *V. fordii* while being consistently upregulated in *V. montana*, which correlated with the resistance of *V. montana* to *Fusarium* wilt. We confirmed that VmWRKY64 can bind to the W-box promoter element of *Vm019719*, enabling *V. montana* to resist *Fusarium* wilt. Remarkably, the deletion of the W-box elements in the promoter of the *Vf11G0978* allele prevented its binding to WRKY64, resulting in the loss of resistance to *Fusarium* wilt in *V. fordii*. These findings not only provide insights into the roles of *NBS-LRRs* in tung tree and their response to *Fusarium* wilt infection but also shed light on the genetic mechanisms underlying resistance to *Fusarium* wilt and the potential application of marker-assisted breeding in tung tree.

## Methods

### Identification of *NBS-LRR* genes

For identification of *NBS-LRRs*, the proteins and coding sequences of *V. montana* and *V. fordii* were obtained from the National Center for Biotechnology Information (NCBI) database (https://www.ncbi.nlm.nih.gov/), as published by Zhang et al. (2019) and Cui et al. (2018) [2, 29]. The hidden Markov models (HMM) for the TIR (PF01582) and NBS (PF00931) domains were obtained from the Pfam (https://pfam.xfam.org/). The NBS-containing sequences were identified in *V. montana* and *V. fordii* using NBS domain by employing HMMER (version 3.0) [55] with an *E*-value of 1e–3, and then these genes were extracted according to their sequencing ID. Among these sequences, the same method was used to determine the TIR-containing sequences. The different types of HMMs of the LRR domain, including LRR1-9, LRV, LRR_adjacent, LRR19-TM, LRRC37, LRRC37AB_C, LRRCT, LRRFIP, LRRNT, and LRRNT_2, were obtained from Pfam and then scanned these LRR domains in NBS-containing sequences in *V. montana* and *V. fordii*. The Paircoil2 (http://cb.csail.mit.edu/cb/paircoil2/paircoil2.html) was used to find the coiled-coil (CC) domains in NBS-containing sequences with a *P*-score cutoff 0.03 [56]. TBtools (version 2.029) [57] was used to determine the chromosomal locations of *NBS-LRRs* in the genomes of both *V. montana* and *V. fordii*. TBtools (version 2.029) [57] was further utilized to compare the chromosomal locations of *NBS-LRRs* between *V. montana* and *V. fordii*.

### Homology in *Vernicia* genomes

The paralogs of *NBS-LRRs* in *V. montana* and *V. fordii*, as well as the orthologs of *NBS-LRRs* between *V. montana* and *V. fordii*, were identified using local BLAST analyses with specific evaluation criteria. These criteria included an *E*-value threshold of ≤ 10–10, identity > 80%, and alignment coverage > 80% [8]. The software TBtools (version 2.029) [57] was utilized to extract the paralogs and orthologs of *NBS-LRRs* pairs, and we then further used this software to calculate the non-synonymous to synonymous per site substitution rates (Ka/Ks), Ks, and Ka values. A Ka/Ks value less than 1 denotes purifying selection, equal to 1 indicates neutral selection, and greater than 1 suggests positive selection [58].

### Gene expression analysis

The RNA-seq data with accession numbers PRJNA445068, PRJNA483508, and PRJNA318350 were obtained from NCBI for the purpose of acquiring the expression levels of *NBS-LRRs* [45]. The RNA-seq data underwent a thorough quality assessment using FastQC (version 0.11.7). Subsequently, low-quality reads and bases were meticulously removed with Trimmomatic (version 0.39) [59]. Finally, the processed reads were aligned using HISAT2 (version 2.2.1) [60] with default parameters, and fragments per kilobase million (FPKM) values were calculated using StringTie (version 2.1.7) [61] with default parameters, following the methodology described by Li et al. [49].

### Yeast one-hybrid (Y1H) assays

To confirm the interactions between the W-box element in the promoter of Vm019719 and WRKY64, we performed Y1H assays using the Matchmaker® Gold Yeast One-Hybrid System (Clontech; TaKaRa). The full-length coding sequence of *WRKY64* was cloned and inserted into the pGADT7. The pAbAi vector underwent modifications to incorporate the sequences of the *Vm019719* promoter. The resulting constructs were linearized using BstBI (New England Biolabs, Beverly, MA, USA) digestion and subsequently transformed into the Y1HGold yeast strain. To determine the minimum inhibitory concentration of Aureobasidin A (AbA) for the pAbAi bait constructions, the transformed yeast strains were cultured on SD medium without uracil. Finally, the pGADT7-WRKY64 construct was introduced into the transformed Y1H yeast strains containing the promoter regions. The interactions between the pAbAi bait constructs and pGADT7-WRKY64 were assessed based on the growth of positive yeast cells on SD/-Ura/AbA medium.

### *Agrobacterium*-mediated VIGS

In accordance with the methodology described by Cao et al. [50], a similar approach was adopted for virus-induced silencing (VIGS) experiments. Gene-specific fragment primers for *WRKY64*, *Vf11G0978*, and *Vm019719* were designed using Primer-BLAST and utilized for cloning and introduction into a pTRV2 vector. Three recombinant plasmids (pTRV2-WRKY64, pTRV2-Vf11G0978, and pTRV2-Vm019719) and control (an empty pTRV2 plasmid) were individually transformed into *Agrobacterium tumefaciens* GV3101. The *Agrobacterium* cells containing pTRV1 or the recombinant plasmids (or pTRV2) were prepared and cultured as described by Cao et al. [50]. The suspensions of the recombinant plasmids (or pTRV2) or pTRV1-containing cells were mixed thoroughly and then inoculated onto the leaves of tung tree plants. Each experiment was carried out at least three times before using the inoculated tung tree plants for functional evaluations after 2 weeks.

### Real-time quantitative PCR (qRT-PCR) assays

Total RNA was extracted from tung tree leaves using the TRIzol reagent (TaKaRa, Tokyo, Japan). The cDNA synthesis was carried out utilizing the PrimerScript™ RT reagent Kit with gDNA Eraser (Takara, Dalian, China). For expression analysis, a qRT-PCR assay was performed in a total reaction volume of 20 μl, which included 2 μl of cDNA template, 10 μl of HieffTM qPCR SYBR® Green Master Mix (Yeasen, Shanghai, China), and 0.2 μl of each primer. The relative gene expression levels were determined using the cycle threshold (Ct) 2^−ΔΔCt^ method. This study included three biological replicates for each sample.

### Supplementary Information

**Additional file 1.** The information of *VfNBS-LRR* genes in *V. fordii*.

**Additional file 2.** The information of *VmNBS-LRR* genes in *V. montana*.

**Additional file 3.** Distribution of various types of *NBS-LRR* on each chromosome in *V. fordii*.

**Additional file 4.** Distribution of various types of *NBS-LRR* on each chromosome in *V. montana*.

**Additional file 5.** Orthologous genes identification between *V. fordii* and *V. montana*.

**Additional file 6.** Duplicated genes identification in *V. montana*.

**Additional file 7.** Selective pressure (Ka/Ks) between paralogous *NBS–LRR* gene pairs in *V. montana*.

**Additional file 8.** Selective pressure (Ka/Ks) between orthologous *NBS–LRR* gene pairs in *V. fordii* and* V. montana*.

**Additional file 9.** All primers used in this study.

## Data Availability

Expression data in this study were available in the SRA database with accession numbers PRJNA445068 [62], PRJNA483508 [63], and PRJNA318350 [64], and other data that support the findings of this study are available in the supplementary material of the article.

## Abbreviations

LRR: Leucine-rich-repeat
NBS: Nucleotide-binding site
Chr: Chromosome
VIGS: Virus-induced silencing
AbA: Aureobasidin A
TIR: Toll/interleukin-1 receptor
TNL: TIR-NBS-LRR
LZ: Leucine zipper
CC: Coiled coil
Ka/Ks: Non-synonymous to synonymous per site substitution rates
FPKM: Fragments per kilobase million
